# Interactive effects of salinity and dietary lipid sources on growth, hepatic lipid metabolism, and transcriptomic profiles in spotted sea bass (*Lateolabrax maculatus*)

**DOI:** 10.3389/fphys.2025.1655953

**Published:** 2025-08-29

**Authors:** Guoxiong Jin, Lu Zhang, Qinghui Ai, Kangsen Mai, Xiaoru Chen

**Affiliations:** ^1^ The Key Laboratory of Mariculture, Ministry of Education, Ocean University of China, Qingdao, China; ^2^ Key Laboratory of Aquatic Nutrition and Smart Farming, Ministry of Agriculture and rural Affairs, Tongwei Agricultural Development Co., Ltd., Chengdu, China

**Keywords:** dietary lipid sources, hepatic lipid metabolism, *Lateolabrax maculatus*, salinity adaptation, transcriptomic analysis

## Abstract

This study investigated the interactive effects of salinity and dietary lipid sources on growth performance, hepatic lipid metabolism, and the underlying molecular mechanisms in spotted sea bass (*Lateolabrax maculatus*). Fish were reared at 0‰ or 20‰ salinities and fed diets containing either fish oil (FO) or soybean oil (SO) for 126 days. Results demonstrated that rearing fish at 20‰ salinity significantly enhanced growth performance but concurrently increased hepatic lipid accumulation compared to rearing at 0‰ salinity. Under the same salinity conditions, dietary lipid sources had no significant effect on fish growth performance, however, compared to FO-based diet the SO-based diet significantly increased hepatic lipid accumulation. Salinity significantly enhanced the growth-promoting effect of SO-based diet, but also aggravated hepatic lipid accumulation in fish. The combination of salinity and FO significantly inhibited lipid synthesis (FAS and ACC activities) and lipolysis (ATGL, MGL activities). RNA-seq identified 9,854 common differentially expressed genes (DEGs). GO enrichment analysis revealed that salinity primarily altered processes related to membrane integrity and energy metabolism, whereas lipid sources regulated organelle structure and fatty acid synthesis. Their interaction regulated catalytic activity and membrane integration processes. KEGG pathway analysis identified salinity-driven shifts in energy/carbohydrate metabolism and lipid-energy sensing, whereas lipid sources dominated fatty acid synthesis. GSEA further highlighted lipid source-dependent regulation of glycerolipid metabolism and unsaturated fatty acid synthesis, alongside salinity-responsive pathways including Ppar signaling and steroid biosynthesis. Key lipid-related genes (*pltp, dgat1, cyp24a1, acadsb*) exhibited differential expression patterns modulated by salinity-lipid interactions. These results support the development of precise nutritional strategies for raising spotted sea bass in varying salinity environments. Replacing FO with SO across salinities is viable when combined with functional additives to regulate lipid metabolism; however, SO inclusion rates should be adjusted downward in seawater to minimize lipid accumulation and optimize performance.

## 1 Introduction

Lipids are essential for fish growth, supplying critical fatty acids and phospholipids, acting as carriers for fat-soluble vitamins, and constituting an indispensable component of aquatic feed (Hardy and Kaushik, 2021). Aquafeeds commonly use fish oil, rich in long-chain polyunsaturated fatty acids (LC-PUFAs), and vegetable oils such as soybean and palm oil, which contain C18 PUFAs. Fish oil (FO) is preferred for its high n-3 LC-PUFA content, good palatability, and favorable n-3/n-6 fatty acid ratio (Alhazzaa et al., 2019; Trushenski and Rombenso, 2020; Hardy and Kaushik, 2021). However, FO resources are limited, and growing market demand has led to continuous price increases. To reduce and eventually eliminate aquaculture’s dependence on FO, researchers have conducted extensive studies on alternative lipid sources over the past 3 decades (Alhazzaa et al., 2019; Hardy and Kaushik, 2021). Plant oils, rich in C18 PUFA, saturated fatty acids (SFA) and monounsaturated fatty acids (MUFA), offer advantages such as lower cost and abundant availability, making them a promising alternative to FO. Numerous studies have demonstrated that partially or completely substituting FO with plant oils does not negatively affect the growth performance of most fish species capable of synthesizing LC-PUFA (Alhazzaa et al., 2019; Mozanzadeh et al., 2021). However, in most marine fish and certain freshwater species, replacing a high proportion of FO with plant oils significantly reduces growth performance and negatively impacts immune health indicators (Alhazzaa et al., 2019; Li et al., 2019; Mozanzadeh et al., 2021). Additionally, during anadromous or catadromous migration, consuming LC-PUFA-rich diets facilitates migratory fish adaptation of to changes in environmental salinity (Rosenlund et al., 2016).

Euryhaline fish can regulate their internal osmotic pressure to adapt to different salinity environments. Changes in salinity significantly impact physiological metabolism and nutritional requirements, particularly by increasing the demand for LC-PUFA. To maintain membrane fluidity and ensure optimal functionality in high-ion-concentration environments, fish must increase the proportion of LC-PUFA, especially docosahexaenoic acid (DHA) and eicosapentaenoic acid (EPA), in their cell membranes (Manuel, et al., 2021). For example, in Atlantic salmon, the DHA content in the gill and intestinal cell membranes is significantly higher during the seawater stage than the freshwater stage (Tocher, 2003). During seawater adaptation, tilapia preferentially mobilizes lipids as an energy source, EPA and ARA may enhance energy efficiency by regulating mitochondrial function (Jiao et al., 2020). Drastic salinity fluctuations can induce oxidative stress and inflammatory responses, where PUFA plays a dual role. DHA and EPA can mitigate salt-induced inflammatory damage by inhibiting the NF-κB pathway, thereby reducing the production of pro-inflammatory factors such as TNF-α (de Souza and Maria, 2010). Additionally, ARA is converted into prostaglandin E2 (PGE2), which regulates Na^+^/K^+^-ATPase activity in low-salinity environments, aiding transmembrane sodium ions transport (Deane and Woo, 2009). Moreover, some studies suggest that the dietary LC-PUFA supplementing is more efficient than relying solely on endogenous synthesis, especially during rapid salinity changes. Salinity significantly affects the fatty acid requirements of euryhaline fish by regulating cell membrane composition, energy metabolism and stress responses. Therefore, feed formulations should consider prevailing salinity, species-specific characteristics, and environmental dynamics to optimize the dietary lipid sources and fatty acid composition.

Spotted sea bass (*Lateolabrax maculatus*), known for its delicious flesh, high nutritional value, rapid growth, and strong disease resistance, exhibits wide tolerance to salinity and temperature. It has become an important economic marine fish along China’s coastal areas, with distinct regional farming practices: seawater net-cage culture in the southeast contrasts sharply with freshwater pond culture in the Pearl River Delta. Although freshwater farming can accelerate growth, it also results in meat quality degradation and reduced flavor, directly affecting market value. In the cultivation of spotted sea bass, a “land-sea” relay farming model is commonly adopted to optimize growth performance and enhance product quality. Specifically, this method entails rearing fish initially in freshwater, followed by transfer to seawater for an additional period of one to 2 months, ultimately contributing to superior overall product quality. Thus, this “growth-quality” trade-off suggests the need to optimize lipid nutrition strategies under varying salinity conditions. This study aimed to systematically analyze the growth performance, fatty acid composition, enzyme activity, and liver transcriptome characteristics of spotted sea bass under different lipid sources (FO vs. soybean oil, SO) and salinity conditions (0‰ vs. 20‰). The goal was to elucidate the molecular mechanisms by which salinity affects lipid metabolism, thereby providing a theoretical basis for developing precise nutritional formulations.

## 2 Materials and methods

### 2.1 Experimental diet

Based on previous studies (Jin et al., 2023; Huang et al., 2024), experimental diets was formulated using FO and SO as lipid sources, creating two diets types with equal nitrogen (45% crude protein) and lipid (15% crude lipid) content. Protein sources, including fishmeal and soybean meal, were processed using an ultra-micro pulverizer (SF-2500, with a fineness of 60 mesh). FO and SO were emulsified using a high-pressure homogenizer (GJB1.5-10, pressure 25 MPa). The “gradient dilution method” mixing process was used: first, ground raw materials and premixes were dry-blended in a twin-shaft paddle mixer (SLHY-500) for 5 min, then, oils were added and wet-mixing for 8 min. The mixture was granulated using a small pellet feed machine (80-type pelletizer, Zhengzhou Kunzhong Machinery Co., Ltd.) to a particle size of 1.5 mm. Feed pellets were dried in hot air at 40°C for 20 min, cooled to room temperature, vacuum-packed, and stored light-proof at 4°C. Composition and nutritional levels of the experimental diets are shown in Supplementary Table S1.

### 2.2 Fish and culture management

Juvenile *L*. *maculatus* were purchased from Zhuhai Qiangsheng Aquatic Products Co., Ltd. The aquaculture trial was conducted at the Tongwei Co., Ltd. Zhuhai Baijiao Base. Prior to the trial, the juveniles were acclimated in net cages for 2 weeks and fed commercial tilapia feed. After acclimatization, 420 healthy juveniles of similar size (89.0 ± 0.5 g) were randomly assigned to 4 treatment groups (D1-D4), with 3 replicate tanks per group (1 m^3^ per tank, 20 fish per tank), and cultured for 126 days. The 12 tanks (1 m^3^ each) were arranged in a randomized block design within the system to minimize environmental gradients (e.g., light, water flow, temperature).

As previous studies suggested that moderate salinity seawater (20‰–25‰) is beneficial for culturing *L. maculatus* (Yang et al., 2021; Hu et al., 2024), experimental fish were reared in systems with salinities of 0‰ and 20‰. Fish in groups D1 and D2 were fed FO and SO-based diets, respectively, at 0‰ salinities conditions. Fish in groups D3 and D4 were fed FO and SO-based diets, respectively, at 20‰ salinities. During the trial, the fish were fed *ad libitum* twice a day (6:00 and 18:00). To maintain water quality (ammonia nitrogen <0.6 mg/L, nitrite <0.2 mg/L), approximately 80% of the tank water is replaced daily with fresh water. Continuous aeration maintained dissolved oxygen levels above 7 mg/L.

### 2.3 Sampling and evaluation of growth performance

At the end of the trial, after a 24-h fast, fish were anesthetized with MS-222 (Sigma, United States), individually weighed and counted. These measurements calculated weight gain rate (WGR), specific growth rate (SGR), survival rate (SR), and feed conversion ratio (FCR). For each tank, 5 fish were randomly selected; body length and weight were measured to calculate the condition factor (CF). Visceral mass and liver were weighed to calculate the viscera-somatic index (VSI) and hepatosomatic index (HSI). Liver tissue was preserved in 4% formaldehyde solution for hematoxylin and eosin (HE) staining and Oil Red staining for histological observation. Intestines were collected for digestive enzyme activity measurement. Additionally, 4 fish per tank were randomly selected, and 1 mL of blood was drawn from the caudal vein using a sterile syringe, placed into a 1.5 mL centrifuge tubes, and stored at 4°C overnight. Blood samples was then centrifuged at 4000 r/min for 10 min, and the supernatant was stored at −80°C for biochemical analysis. Liver tissue was divided into three parts, each placed in a 1.5 mL enzyme-free centrifuge tube, snap-frozen in liquid nitrogen, and stored for transcriptome analysis, enzyme activity assays, and gene/protein expression analysis. Furthermore, 4 additional fish were collected, with 2 fish used to measure body composition.

The growth performance and morphological parameters measured in this experiment included: weight gain rate (WGR), specific growth rate (SGR), survival rate (SR), feed conversion ratio (FCR), condition factor (CF), viscera-somatic index (VSI), and hepatosomatic index (HSI). The above parameters were calculated using standard formulas as reported.

### 2.4 Approximate composition analysis

Dietary approximate composition was analyzed according to the methods outlined by AOAC (1995) (Feldsine et al., 2002). Feed moisture was determined using the 105°C constant temperature drying method. Crude protein content was measured using a Leco FP-528 automatic protein analyzer. Crude lipid content was determined using an OPSIS SX-360 crude lipid analyzer. Crude ash content was measured using the 550°C muffle furnace ignition method.

### 2.5 Measurement of intestinal digestive enzyme activity

Intestinal tissues from three fish per tank (biological replicates) were homogenized, and the activities of α-amylase, trypsin, and lipase were measured using commercial kits. The digestive enzyme kits were purchased from Nanjing Jiancheng Bioengineering Institute (Nanjing, China). Each sample was assayed in duplicate (technical replicates). Detailed experimental procedures are provided in the kit instruction manuals.

### 2.6 Measurement of liver lipid metabolic enzyme activity

Liver samples from three fish per tank (biological replicates) were analyzed. Liver enzyme activities were determined using enzyme-linked immunosorbent assay (ELISA). Lipid metabolism-related indicators, including alanine aminotransferase (ALT), aspartate aminotransferase (AST), malate dehydrogenase (MDH), alkaline phosphatase (ALP), and lipoprotein lipase (LPL), were measured using kits from Nanjing Jiancheng Bioengineering Institute. The activities of Δ6 Fads2, Δ5 Fads2, triglyceride lipase (ATGL), fatty acid synthase (FAS), acetyl-CoA carboxylase (ACC), ATP-citrate lyase (ACL), 3-phosphoglycerate dehydrogenase (PHGDH), and monoacylglycerol lipase (MGL) were determined using kits from Shanghai Jianglai Biological Co. All measurements were performed in duplicate (technical replicates) per sample.

### 2.7 Hepatic histological analysis

The liver samples fixed in 4% paraformaldehyde (3 fish/tank) were frozen and sectioned. Neutral lipids were stained with Oil Red O (Direct Red 80; Aldrich, Milwaukee, WI, United States) as previously described (Jin et al., 2023). The relative area of lipid droplets in the Oil Red O-stained was analyzed using Image-Pro Plus 6.0 software (Media Cybernetics, Inc., Rockville, MD, United States) by calculating the ratio of total red lipid droplet area to total field area per field. All image data presented represent the samples from three random fields in each section.

### 2.8 Hepatic transcriptome sequencing

Total RNA was extracted from liver tissue of three biological replicates per tank using TRIzol reagent (Life Technologies, United States). The quality and quantity of extracted total RNA were determined using agarose gel electrophoresis, NanoDrop spectrophotometry, and an Agilent 2100 Bioanalyzer (Agilent Technologies, Palo Alto, CA, United States). Total RNA was enriched using oligo (dT) beads. High-quality RNA samples were used to construct sequencing libraries using the Illumina HiSeqTM 2500 platform at Gene *Denovo* Biotechnology Co., Ltd. (Guangzhou, China). Raw reads were quality-filtered using fastp (v.0.18.0) to obtain high-quality clean reads. After removing rRNA using the short-read aligner Bowtie2 (version 2.2.8), the remaining reads were mapped to the reference genome using TopHat2 (version 2.1.1).

Transcript expression levels were quantified as FPKM (Fragments Per Kilobase of transcript per Million mapped reads). Differentially expressed genes (DEGs) were identified using the edgeR package (v. 3.12.1) (http://www.r-project.org/). DEGs with a fold change (FC) ≥ 2 and a false discovery rate (FDR) adjusted P-value <0.05 were considered significant. All DEGs were mapped to the selected GO database (http://www.geneontology.org/). KEGG (Kyoto Encyclopedia of Genes and Genomes) enrichment analysis was performed using the KOBAS web server (http://kobas.cbi.pku.edu.cn/). A corrected *P* < 0.05 was set as the threshold for statistical significance in GO and KEGG enrichment analyses. Several differentially expressed genes were selected for RT-PCR analysis to measure their relative expression levels, and the results were compared with the transcriptome data to validate the reliability of the transcriptome findings.

### 2.9 Real-time quantitative PCR analysis

Total RNA extration and cDNA synthesis were performanced using liver samples, following our previous procedure (Jin et al., 2023). Quantitative real-time PCR (q-PCR) was conducted to determine the relative mRNA expression levels of genes involved in hepatic lipid metabolism. The specific primers used for qPCR are listed in Supplementary Table S2. qPCR reactions were conducted as previously described (Jin et al., 2023) Relative mRNA expression levels were calculated using the 2^−ΔΔCT^ method, with β-actin as the reference gene. Each sample was analyzed in triplicate.

### 2.10 Statistical methods

Prior to analysis, data homogeneity and variance equality were tested using SPSS 20.0 software. Data following a normal distribution proceeded to further analysis. Data are presented as mean ± standard error, and analyzed using two-way analysis of variance (ANOVA) in SPSS 22. The 2 × 2 factorial design were analyzed using the general linear model (GLM) procedure. Two-way ANOVA assessed the significance of the main effects (salinity, lipid source) and their interaction (significance level *P* < 0.05). Where the interaction was significant (*P* < 0.05), pairwise comparisons between groups were conducted using Tukey’s HSD test. Where the interaction was not significant, the main effects were interpreted, and independent samples t-tests were used to compare salinity effects within each lipid source, and lipid source effects within each salinity level.

## 3 Results

### 3.1 Growth performance and morphological indicators

As shown in Table 1, compared to fish reared at 0‰ salinity, those at 20‰ salinity exhibited a 9.88% increase in WGR (*P* = 0.057) and a 5.79% increase in SGR (*P* = 0.072), while the FCR decreased by 3.19% (*P* = 0.131). Compared to the SO groups, the FO groups had higher FCR (*P* = 0.023) and lower HSI (*P* = 0.032), while no significant differences were observed in the other induces (*P* > 0.05). Two-way ANOVA indictated that the sanlity × lipid source interaction did not significantly affected the growth performance and morphological indices (*P* > 0.05).

**TABLE 1:** Growth performance and morphological indices of Spotted sea bass among the different groups.

Parameter	Groups				P Value of two-way analysis of variance		
	D1	D2	D3	D4	Salinity	Lipid sources	Salinity X lipid sources
WGR	507.85 ± 9.60	511.35 ± 11.92	523.98 ± 13.92	539.62 ± 3.89	0.057	0.381	0.575
SGR	1.26 ± 0.01	1.27 ± 0.02	1.28 ± 0.02	1.31 ± 0.01	0.072	0.403	0.591
FCR	0.94 ± 0.01	0.95 ± 0.01	0.92 ± 0.00	0.95 ± 0.01	0.131	0.023	0.350
SR	98.75 ± 1.25	97.50 ± 1.44	98.81 ± 1.19	97.56 ± 1.41	0.965	0.365	1.000
CF	1.92 ± 0.02	1.86 ± 0.03	1.92 ± 0.02	1.93 ± 0.02	0.283	0.368	0.247
HSI	12.33 ± 0.44	11.51 ± 0.41	12.23 ± 0.37	11.29 ± 0.18	0.661	0.032	0.882
VSI	1.33 ± 0.05	1.28 ± 0.03	1.45 ± 0.02	1.42 ± 0.07	0.064	0.853	0.677

Note: values are mean ± SEM (n = 3, three fish per tank). Values in the same line with different superscripts are significantly different (*P* < 0.05).

IBW: initial body weight, FBW: final body weight, WGR: weight gain rate, SGR: specific growth rate, FCR: feed conversion ratio, SR: survival rate, CF: condition factor, HSI: hepatosomatic index, VSI: viscerosomatic index.

### 3.2 Liver tissue morphological structure

**FIGURE 1:**
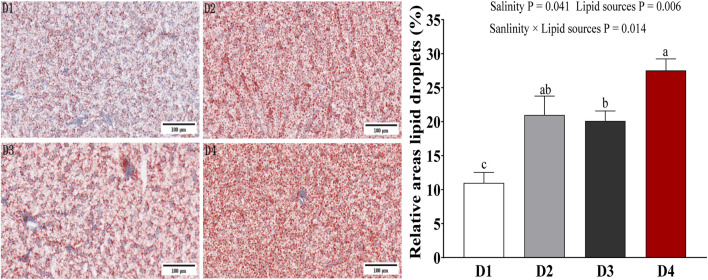
Liver tissue structure of Spotted sea bass among the different groups. Note: values are mean ± SEM (n = 3, three fish per tank). Histological metrics (lipid droplet areas) were quantified for statistical comparison, and groups marked with different lowercase letters indicate significant differences at *P <* 0.05 as determined by two-way ANOVA followed by a *post hoc* test. These differences reflect statistically distinct liver tissue characteristics between experimental groups. Groups D1 and D2 were fed FO- and SO-based diets, respectively, under freshwater conditions; groups D3 and D4 received the same diets under seawater conditions.

Oil Red O staining (Figure 1) showed that liver cells ingroups D2-D4 exhibited varying degrees of lipid infiltration, increased lipid droplets vacuolization, and nuclear displacement, indicating nutritional fatty liver. Compared to fish reared at 0‰ salinity, the liver lipid droplet area was relatively higher in fish maintained at 20‰ salinity, especially in the two FO groups (*P* = 0.041). SO groups also had a significantly higher lipid droplet area than the FO groups (*P* = 0.006). The interaction between lipid source and salinity significantly influenced the liver lipid droplet area of fish (*P* = 0.014).

### 3.3 Intestinal digestive enzyme activity in fish from different treatment groups

As shown in Table 2, compared to the 0‰ groups, the 20‰ groups had higher activities of trypsin (*P* = 0.004) and amylase (*P* < 0.001), with the highest enzymatic activity in group D3. Group D4 also showed significantly higher lipase activity than group D2 (*P* = 0.041). Two-way ANOVA indicated that the dietary lipid sources had a significant effect on lipase (*P* < 0.001) and trypsase (*P* = 0.044) activity in fish, but no significant effects were observed on amylase activities (*P* = 0.737). The interaction between lipid source and salinity significantly influenced the lipase (*P* = 0.001) and amylase (*P* = 0.007) activities of fish.

**TABLE 2:** Activities of intestinal digestive enzymes of Spotted sea bass among the different groups.

Enzyme	Groups				P Value of two-way analysis of variance		
	D1	D2	D3	D4	Salinity	Lipid sources	Salinity X Lipid sources
Trypsase	408.03 ± 97.75	421.74 ± 78.6	1035.93 ± 74.11	517.41 ± 87.92	0.004	0.044	0.232
Lipase	2.98 ± 0.19	2.09 ± 0.31	2.41 ± 0.32	3.45 ± 0.17	0.041	<0.001	0.001
Amylase	0.93 ± 0.06	0.98 ± 0.02	1.20 ± 0.10	1.14 ± 0.07	0.001	0.737	0.007

Note: values are mean ± SEM (n = 3, three fish per tank). Values in the same line with different superscripts are significantly different (*P* < 0.05).

### 3.4 Liver lipid metabolism-related enzyme activity

**FIGURE 2:**
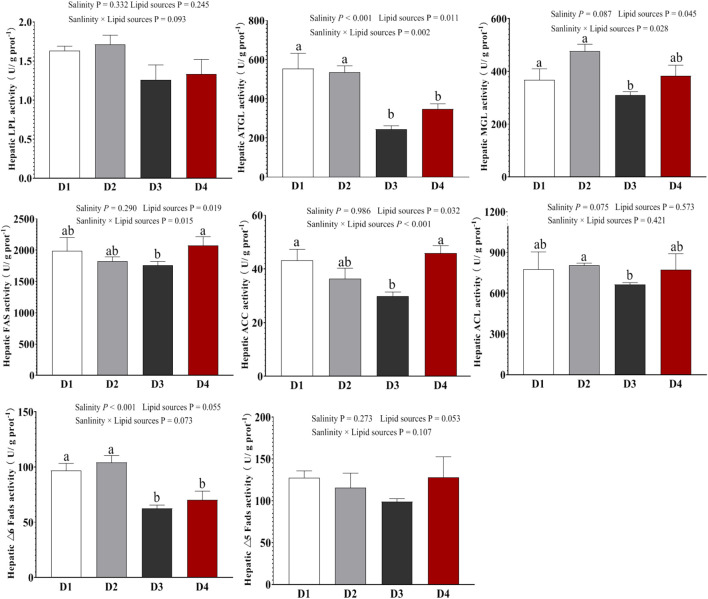
Enzyme activities associated with lipid metabolism in the liver of Spotted sea bass among the different groups. Note: values are mean ± SEM (n = 3, three fish per tank), and different letters on bars represented significant differences among the different groups (*P* < 0.05).

Liver lipid metabolism-related enzyme activities are shown in Figure 2. Compared to the 0‰ groups, the 20‰ groups exhibited significantly lower activities of enzymes related to lipolysis (ATGL and MGL) and unsaturated fatty acid biosynthesis (△6 Fads) (*P* < 0.001). No significant differences were observed in those above induces between the FO and SO groups (*P* > 0.05). Additionally, the D3 group showed the relatively low FAS, ACC, and ACL enzyme activities than those in the D1, D2, and D4 groups. No significant differences in LPL and △5 Fads enzyme activities were observed between the groups (*P* > 0.05). Two-way ANOVA showed that the interaction between lipid source and salinity had no significant effect on liver LPL, ACL, or △6Fads enzyme activities (*P* > 0.05), but it significantly affected the activities of ATGL, MGL, FAS and ACC enzymes (*P* < 0.05).

### 3.5 Hepatic transcriptome profiling

After filtering and quality control of the RNA-Seq data, 587,439,562 raw reads and 88,115,934,300 raw bases were obtained for the four groups. This yielded 586,333,854 clean reads and 87,389,632,639 clean bases, with clean data ratios >99.76% for all samples. Intra-group sample reproducibility, assessed by pairwise sample correlation coefficients based on FPKM values, exceeded 95%, indicating good reproducibility (Supplementary Figure S1).

The Venn diagram (Supplementary Figure S2A) showed 9,854 differentially expressed genes (DEGs) common to all four groups (expression threshold >1 FPKM). Principal component analysis (PCA) (Supplementary Figure S2B) revealed that D1 was distantly clustered from D3 but was closer to D2. D3 and D4 were more similar to each other.

For pairwise comparisons of the treatment groups based on sample relationships (Supplementary Figure S2C), the number of significantly differentially expressed genes (DEGs) for each comparison was as follows: D1 vs. D2: 342 DEGs (176 upregulated, 166 downregulated); D1 vs. D3: 1,267 DEGs (700 upregulated, 567 downregulated); D1 vs. D4: 966 DEGs (449 upregulated, 517 downregulated); D2 vs. D3: 534 DEGs (289 upregulated, 245 downregulated); D2 vs. D4: 420 DEGs (177 upregulated, 243 downregulated); D3 vs. D4: 183 DEGs (78 upregulated, 105 downregulated). These results indicate substantial differences in gene expression among the treatment groups, emphasizing the influence of various factors on Spotted sea bass gene expression.

### 3.6 Differential gene GO and KEGG analysis

**FIGURE 3:**
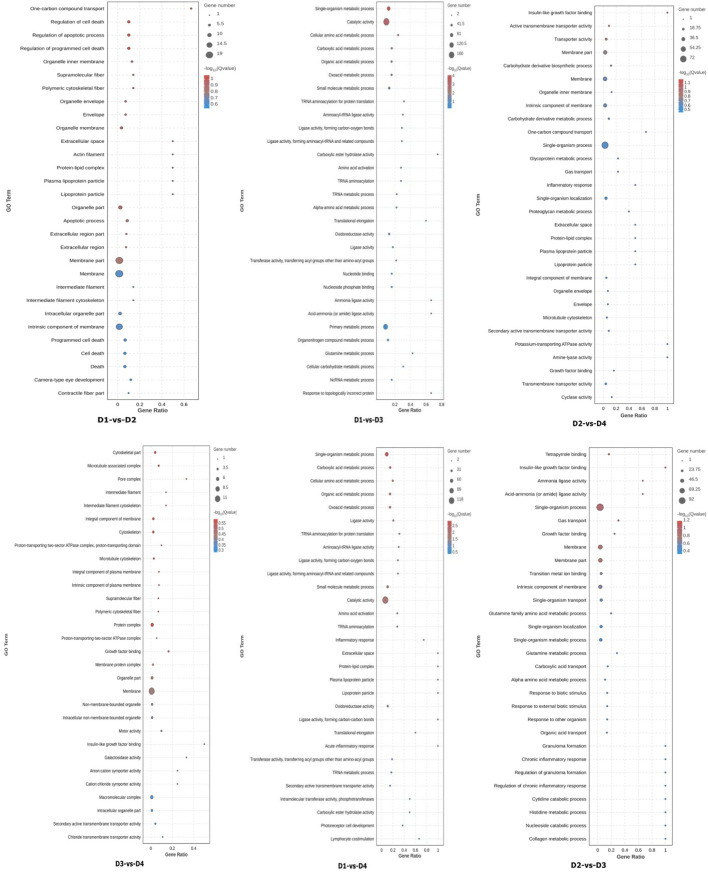
Enrichment analysis of liver differentially expressed genes (DEGs) gene ontology (GO) among different groups. Note: Groups D1 and D2 were fed FO- and SO-based diets, respectively, under freshwater conditions; groups D3 and D4 received the same diets under seawater conditions.

All DEGs were mapped to the Gene Ontology (GO) database for enrichment analysis (Figure 3). Comparing different salinity levels within the same lipid source (e.g., D1 vs. D3, D2 vs. D4), salinity primarily influenced GO terms related to: intrinsic component of membrane, oxidoreductase activity, oxoacid metabolic process. When comparing different lipid sources under the same salinity (e.g., D1 vs. D2, D3 vs. D4), differences in lipid sources mainly affected the following processes: organelle part, membrane part, cytoskeleton, protein complex. The interaction between salinity and lipid source mainly influenced the following processes: catalytic activity, integral component of membrane.

**FIGURE 4:**
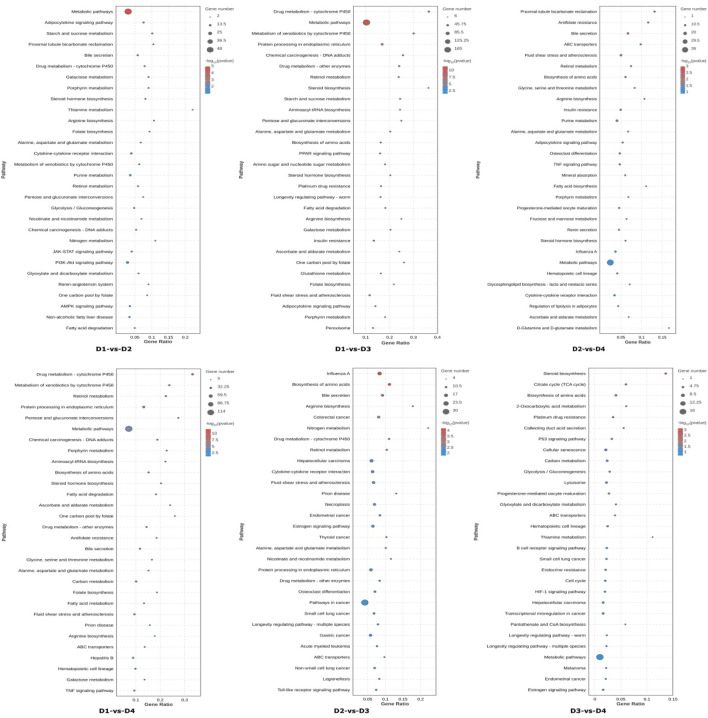
The top 30 significantly enriched KEGG pathways of differentially expressed genes (DEGs). Note: The pathways are shown in the vertical axis. The dot size indicates the number of genes and the color indicates the q-value. Groups D1 and D2 were fed FO- and SO-based diets, respectively, under freshwater conditions; groups D3 and D4 received the same diets under seawater conditions.

Then DEGs were mapped to the KEGG (Kyoto Encyclopedia of Genes and Genomes) database (Figure 4). By comparing different salinity groups under the same lipid source (D1 vs. D3, D2 vs. D4), salinity changes significantly regulated the following metabolic pathways: energy metabolism and carbohydrate utilization, lipid metabolism and energy sensing. By comparing different lipid sources under the same salinity (D1 vs. D2, D3 vs. D4), differences in lipid sources significantly affected the following metabolic pathways: fatty acid metabolism and lipid synthesis, bile secretion. The interaction between salinity and lipid source primarily influenced the following pathways: carbon metabolism, regulation of lipolysis, ABC transporter pathways. These results suggest that both salinity and lipid source have significant effects on various biological processes and metabolic pathways, and their interaction further influences certain key cellular and metabolic functions.

### 3.7 Differential analysis and mining key genes related to specific phenotypes

**FIGURE 5:**
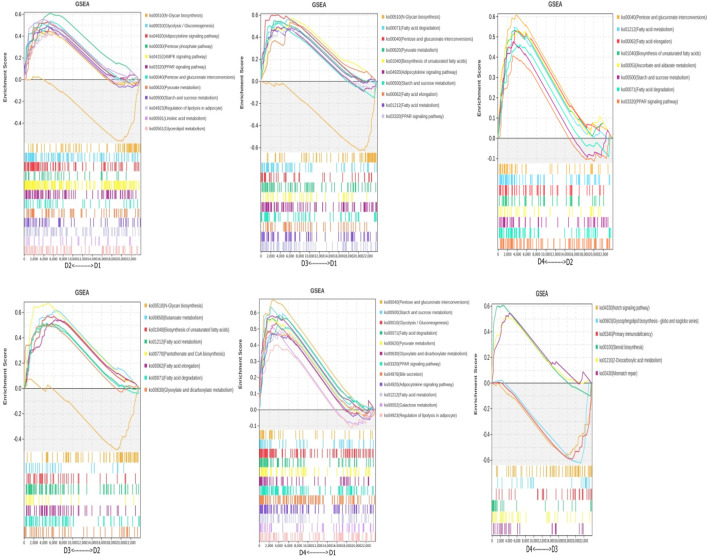
Differential metabolic pathways were obtained using GSEA. Note: Groups D1 and D2 were fed FO- and SO-based diets, respectively, under freshwater conditions; groups D3 and D4 received the same diets under seawater conditions.

Using the expression data of all genes, the genes and performed Gene Set Enrichment Analysis (GSEA) were reannotated (Figure 5). The results identified the following differential metabolic pathways: between different lipid sources under the same salinity (D1 vs. D2, D3 vs. D4): glycerolipid metabolism, unsaturated fatty acid synthesis, and regulation of lipolysis in adipocytes. Between different salinity levels under the same lipid source (D1 vs. D3, D2 vs. D4), several key metabolic pathways were identified, including fatty acid degradation, steroid biosynthesis, unsaturated fatty acid biosynthesis, and Ppar signaling pathway. Interaction between salinity and lipid source (D1 vs. D4, D2 vs. D3): Ppar signaling pathway and starch and sucrose metabolism.

Differentially expressed genes associated with lipid metabolism were selected and identified, and functional pathway analysis was performed. Common DEGs related to lipid metabolism were systematically selected from the pairwise comparisons. Key pathways and genes involved in lipid metabolism were identified using Gene Set Enrichment Analysis (GSEA). These two gene sets were then matched to select representative genes that were highly associated with lipid metabolism (Supplementary Table S3). Comparing different lipid sources within the same salinity, *pltp* mRNA levels showed no significant changes between D1 vs. D2, but were upregulated in in D3 vs. D4 (Log2FC = 1.46) and further upregulated D1 vs. D3 (Log2FC = 1.62). The expression levels of *dgat1* were upregulated in D2 vs. D3 (Log2FC = 1.11) and continuously upregulated in D2 vs. D4 (Log2FC = 1.16). Those results indicated that the expression levels of *pltp* and *dgat1* are significantly influenced by the dietary lipid source. Comparing different salinity within the same lipid source, *cyp24a1* transcripts were significantly upregulated in D1 vs. D3 (Log2FC = 2.23) and remained upregulated in D2 vs. D4 (Log2FC = 1.45). The transcripts of *acadsb* were downregulated in both D1 vs. D3 (Log2FC = −1.38) and D2 vs. D4 (Log2FC = −1.28), suggesting that *cyp24a1* and *acadsb* are key genes involved in salinity adaptation. Analysis of differential genes related to the interaction between salinity and lipid source showed that *hsd17b7* mRNA levels were significantly upregulated in D1 vs. D2 (Log2FC = 1.55) and further upregulated in D1 vs. D3 (Log2FC = 2.37), but downregulated in D3 vs. D4 (Log2FC = −1.29), indicating that its expression is influenced by both salinity and lipid source under high salinity conditions. The mRNA levels of *cyp2r1* were downregulated in D1 vs. D3 (Log2FC = −2.85) and upregulated in D3 vs. D4 (Log2FC = 2.20), indicated that the lipid source may reverse the effect of salinity on *cyp2r1* expression. The transcripts of *acadl* were significantly downregulated in D1 vs. D2 (Log2FC = −2.32) and D1 vs. D3 (Log2FC = −1.66), but further downregulated in D3 vs. D4 (Log2FC = −1.90), indicating that its expression is co-regulated by salinity and lipid source. The mRNA levels of *acsl4* were upregulated in both D1 vs. D3 and D1 vs. D4 (Log2FC = 1.54 and 1.25), but showed no significant changes in D3 vs. D4, suggesting that it may be co-regulated by salinity and lipid source.

### 3.8 qRT-PCR validation

**FIGURE 6:**
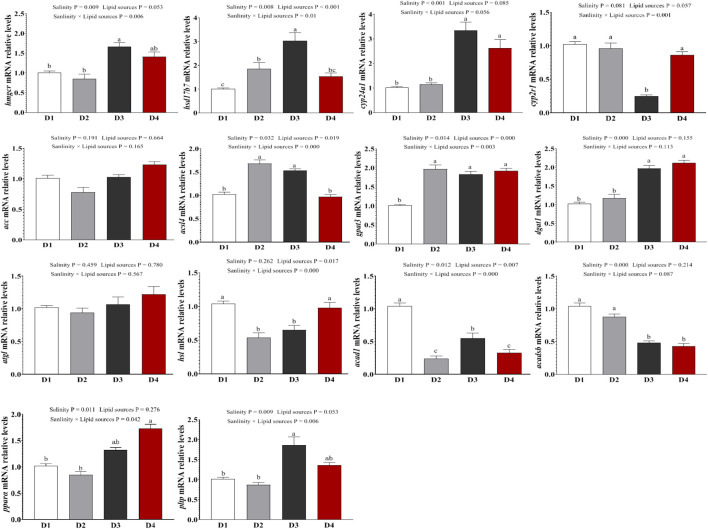
Expression levels of genes related to steroid synthesis, lipid synthesis and decomposition metabolism in the liver of fish in each treatment group, as well as the PPAR signaling pathway. Note: values are mean ± SEM (n = 3, three fish per tank), and different letters on bars represented significant differences among the different groups (*P <* 0.05).

To verify the reliability of the transcriptome profile of key genes, genes related to steroid metabolism (*hmgcr, hsd17b7, cyp24a1, cyp2r1*), lipid synthesis metabolism (*acc, dgat1, acsl4, gpat3*), lipid degradation metabolism (*atgl, hsl, acad1, acadsb*), and the Ppar signaling pathway (*pltp, ppara*) were selected for qPCR confirmation (Figure 6). For steroid metabolism-related gene expression (Figure 6A), compared to the 0‰ groups, the expression of *hmgcr* (*P =* 0.009) and *cyp24a1* (*P =* 0.081) in the fish was significantly upregulated in the 20‰ groups. The expression of *hsd17b7* (*P =* 0.01) and *cyp2r1* (*P =* 0.056) was influenced by the interaction between salinity and lipid source. For lipid synthesis metabolism-related gene expression (Figure 6B), compared to the 0‰ groups, *dgat1* expression was significantly upregulated in the 20‰ groups (*P <* 0.001), with a trend of upregulation in acc; whereas *acsl4* (*P <* 0.001) and *gpat3* (*P =* 0.003) expression was influenced by the interaction between salinity and lipid source. For lipid degradation metabolism-related gene expression (Figure 6C), *acadsb* expression was significantly downregulated the 20‰ groups compared to the 0‰ groups (*P <* 0.001). The expression levels of *hsl* and *acad1* were influenced by the interaction between salinity and lipid source (*P <* 0.001). Salinity significantly influenced the expression of pltp (P = 0.009) and ppara (P = 0.011) in the Ppar signaling pathway, with both genes showing higher expression in the 20‰ groups than those of 0‰ groups (Figure 6D).

## 4 Discussion

The present study systematically investigated the interactive effects of dietary lipid sources and salinity on spotted sea bass, revealing critical trade-offs between growth enhancement and hepatic lipid metabolism. Plant oils are widely used in aquafeeds to reduce reliance on FO, but their effectiveness depends on species-specific LC-PUFA biosynthesis capacity and dietary fishmeal level (Mozanzadeh et al., 2021; Hardy and Kaushik, 2021). For example, in studies conducted on the weak LC-PUFA-synthesizing species “*Trachinotus ovatus*,” it was observed that the exclusion of fishmeal, coupled with the complete substitution of FO with SO, led to a significant reduction in growth performance and feed efficiency (Wang et al., 2020). However, when the diet included 25% fishmeal, plant-based oils such as tea tree oil and perilla oil were capable of fully substituting for FO without adversely impacting growth performance or feed efficiency ratios (Guo et al., 2021). Yan et al. (2020) reported that in hybrid grouper (*Epinephelus fuscoguttatus* × *E. lanceolatus*), the inclusion of 43% fishmeal allowed for the complete substitution of FO with plant-based oils such as corn oil, olive oil, and rapeseed oil, while still supporting normal growth. However, when 40% white fishmeal was used, the replacement of FO with SO, rapeseed oil, and palm oil resulted in a significant reduction in growth performance (An et al., 2020). Under exclusively plant-based feed conditions, certain freshwater fish species with the capacity for LC-PUFA biosynthesis, as well as herbivorous marine fish, are capable of utilizing EFA derived from plant oils to sustain normal growth (Mozanzadeh et al., 2021; Hardy and Kaushik, 2021). Consistent with this, under 25% fishmeal conditions, we observed no significant differences in growth performance or feed utilization between the SO and FO dietary groups. Similarly, under 20% fishmeal conditions, the complete replacement of FO with tea tree oil, perilla oil, or SO was found to adequately support normal growth in Spotted sea bass (Jin et al., 2023). However, when 25% defatted fishmeal was used in the feed, replacing FO with rapeseed oil and purified ALA oil did not significantly negatively impact the growth performance of s*potted sea bass*, but the specific growth rate decreased by 7.18%–13.84% (Xu et al., 2016). These results indicate that Spotted sea bass possesses a certain capacity for synthesizing LC-PUFA. Furthermore, when a specific amount of fishmeal is incorporated into the feed, plant-based oils can effectively substitute for dietary FO in supproting growth.

Beyond growth support, LC-PUFA-rich FO plays a role in salinity adaptation in euryhaline fish. In low-salinity environments, providing fish with a diet that contains appropriate levels of LC-PUFA can enhance ion reabsorption and osmotic pressure regulation in black seabream, thereby supporting their normal growth and physiological functions (Bao et al., 2022). Under low salinity conditions, the liver LC-PUFA synthesis capacity of rabbitfish (*Siganus canaliculatus*), tilapia, thicklip grey mullet (*Chelon labrosus*) increases to aid adaptation to the low-salinity environment (Xie et al., 2015; Marrero et al., 2024). This study revealed that, under freshwater conditions, the FO diets did not provide a significant additional growth advantage for spotted sea bass compared to the SO diets. Potential explanations include: 1) Spotted sea bass has an inherent capacity to synthesize LC-PUFAs, and at 0‰ salinity, hepatic △6 Fads enzyme activity is markedly increased, promoting endogenous LC-PUFA synthesis to fulfill normal growth requirements (Xie et al., 2022). Further investigation into tissue fatty acid composition is warranted. 2) Under acute salinity stress, fish may exhibit an increased demand for LC-PUFA (Zhang et al., 2017; Dong et al., 2020). However, during long-term aquaculture trials, fish may potentially modulate their physiological metabolism, including cholesterol metabolism, phospholipid metabolism, and fatty acid metabolism, to adapt to freshwater conditions and thereby reduce their reliance on LC-PUFA (Zhang et al., 2017; Dong et al., 2020; Bao et al., 2022). This hypothesis is consistent with our observed upregulation of endogenous LC-PUFA synthesis genes (e.g., △6 Fads) and requires further validation. These adjustments might contribute to the successful freshwater aquaculture of spotted sea bass.

The liver, a central hub of lipid metabolism, is highly sensitive to both dietary lipid source and salinity. Disruption may lead to excessive lipid accumulation, adversely affecting hepatic structure and function (Mozanzadeh et al., 2021; Liu et al., 2021; Chen et al., 2023). In the present study, under identical salinity, no significant difference in the HSI was observed between the FO and SO groups. However, the FO groups exhibited significantly larger liver lipid droplet areas compared to the SO group. Similarly, Jin et al. (2023) further demonstrated that replacing FO with various plant oils in the diet had no significant impact on the HSI of spotted sea bass, however, the liver lipid droplet area in the plant oil groups was significantly larger compared to that in the FO group. In hybrid grouper, replacing FO with plant oils in the diet did not significantly affect the HSI. In the FO group, liver cells were normal with few lipid droplets, no swelling, and visible nuclei. In all plant oil groups, liver cells showed severe vacuolization and were filled with lipid droplets (Yan et al., 2020). Additionally, in the FO diet group, the liver lipid droplet area of the spotted sea bass was significantly greater under 20‰ salinity conditions than under 0‰ salinity conditions. In studies on large yellow croaker (*Larimichthys crocea*), low salinity stress was found to inhibit the liver’s ability to absorb and metabolize lipid, leading to disrupted lipid metabolism and lipid accumulation in the liver (Liu et al., 2021). Fan et al. (2022) alos found that the high salinity stress induce the expression of genes related to fatty acid synthase in the hepatopancreas of oriental river prawn (*Macrobrachium nipponense*). The present study also found that compared to 20‰ salinity groups conditions, 0‰ salinity groups significantly reduced the activity of intestinal trypsin and amylase in *spotted sea bass*, but increased the activity of liver lipid metabolism enzymes (LPL, ATGL, and MGL). These findings suggest that under varying salinity conditions, euryhaline fish can adjust by affecting the digestion and absorption of nutrients, increasing the synthesis of liver triglycerides and cholesterol to meet the energy demands for osmoregulatory processes and adapt to changes in salinity (Sheridan, 1988; Si et al., 2018; Bao et al., 2022). Importantly, these enzymatic changes align with our transcriptomic data: The observed upregulation of lipid catabolism genes (e.g., *acadsb* in FO groups) under 20‰ salinity conditions correlates with lower hepatic LPL/ATGL/MGL activity, while their downregulation in SO/freshwater groups corresponds to elevated enzyme activity. This suggests post-transcriptional compensation may occur when gene expression is suppressed.

Transcriptome analysis further provided deeper mechanistic insights into how dietary lipid source and salinity influenced the hepatic lipid phenotypes observed in *spotted sea bass*. Both salinity and dietary lipid source significantly altered the expression of numerous genes (salinity levels: 183–342 DEGs; lipid sources: 420–1267 DEGs), predominantly enriched in key lipid metabolic pathways, including energy metabolism, PPAR signaling pathway, adipocytokine signaling pathway, fatty acid degradation/synthesis, and bile secretion. This aligns with previous findings in spotted sea bass (Jin et al., 2023) and other euryhaline species like tilapia, spotted grouper (*Scatophagus argus*), and black seabream (*Acanthopagrus schlegelii*), where steroid biosynthesis, glycerolipid metabolism, fatty acid pathways, and PPAR signaling are central to salinity adaptation and lipid metabolism (Xu et al., 2015; Chen et al., 2023; Bao et al., 2022). GSEA analysis in our study specifically highlighted the impact on steroid biosynthesis, glycerolipid metabolism, fatty acid synthesis/degradation, and Ppar signaling pathways in the liver.

Integrating DEG expression and GSEA results revealed specific mechanistic links: 1) hepatic lipid accumulation under FO/SO: The observed increase in liver lipid droplet area under FO (vs. SO) and especially under 20‰ salinity (vs. 0‰ salinity) conditions with FO correlates mechanistically with the upregulation of key genes involved in cholesterol synthesis (*hmgcr, cyp24a1*), fatty acid/lipid synthesis (*acc, dgat1*), and the Ppar signaling pathway (*pltp, pparα*) in these groups. Upregulation of *pparα* and its targets like *pltp* is known to promote lipid uptake, storage, and potentially reduce oxidation, contributing to lipid accumulation. Similarly, the upregulation of lipid synthesis genes (*dgat1, gpat3*) and downregulation of lipid catabolism genes (*acad1, acadsb*) in SO groups compared to FO, particularly in the 0‰ salinity groups, provides a direct molecular explanation for the hepatic lipid accumulation observed with plant oil-based diets, as seen in Jin et al. (2023). 2) Steroid biosynthesis and salinity adaptation: The upregulation of steroid biosynthesis genes (*cyp2r1, cyp24a1, hsd17b7*) in seawater compared to freshwater, especially under FO, suggests an increased demand for cholesterol and its derivatives for osmoregulatory processes (e.g., membrane fluidity adjustment, hormone synthesis) in higher salinity. This mirrors findings in black seabream where low salinity induced hmgcr for cholesterol synthesis for energy (Bao et al., 2022). The contrasting downregulation of steroid genes in some species under low salinity (Chen et al., 2023; Liu et al., 2021) highlights species-specific strategies, but underscores the critical role of this pathway in salinity response. 3) Ppar signaling as a central integrator: The consistent enrichment and differential expression within the Ppar signaling pathway (*pparα, pltp*) across both dietary and salinity factors strongly implicate it as a key transcriptional regulator orchestrating the hepatic metabolic response. Pparα activation typically promotes fatty acid oxidation, but its specific effects can be context-dependent and influenced by ligand availability (dietary fatty acids). Our data suggests its involvement in mediating the lipid storage phenotype observed under different conditions.

Therefore, coordinated regulation of hepatic lipid metabolic pathways—particularly steroid biosynthesis for osmoregulation, fatty acid synthesis/catabolism, and Ppar signaling as a master regulator—appears crucial for spotted sea bass to adapt its lipid homeostasis to varying salinity and lipid sources. These mechanistic insights directly inform precision feed formulation for spotted sea bass aquaculture under salinity variation. In freshwater systems, where SO-induced lipid accumulation is pronounced, strategically reducing dietary SO levels (e.g., partial replacement with low-n-6 PUFA oils like palm oil) or supplementing Ppar agonists (e.g., fibrates) could mitigate hepatic steatosis while maintaining growth benefits (Zhang et al., 2021; Fan et al., 2024; Chen et al., 2025). In dynamic salinity environments (e.g., “land-sea” relay farming model), real-time monitoring of salinity shifts should trigger adaptive feeding protocols: increasing FO proportion during acute salinity stress to support LC-PUFA-dependent osmoregulation (Zhang et al., 2017; Dong et al., 2020), and reverting to SO-based diets in stable phases to reduce feeding costs (Nasopoulou and Zabetakis, 2012; Sang et al., 2025).

However, this study investigated the interactive regulatory mechanisms of salinity and dietary lipid sources on growth and hepatic lipid metabolism in spotted sea bass, we acknowledge several limitations. First, the relatively short trial duration of 126 days may not fully reflect long-term adaptive responses; future studies could extend observation periods to capture the long-term effects. Second, although fish were statistically randomized across tanks to minimize tank effects, unmeasured environmental stressors could potentially influence metabolic outcomes. Finally, our controlled laboratory conditions—while essential for mechanistic insight—differ from commercial aquaculture settings where dynamic salinity fluctuations and higher stocking densities occur. To address these aspects, future research should: 1) Validate the key salinity-lipid interaction pathways (Ppar signaling, steroid biosynthesis, etc.) identified in spotted sea bass across other euryhaline species like Nile tilapia and Atlantic salmon to determine evolutionary conservation. 2) Conduct translational studies in commercial-relevant environments that simulate dynamic salinity regimes and practical feeding protocols. Such work is critical to assess the growth performance, lipid deposition patterns, and economic viability (e.g., cost-benefit analysis) of optimized strategies—such as soybean oil-based diets supplemented with Ppar agonists—for reducing fish oil reliance in field applications.

## 5 Conclusion

This study systematically investigates the regulatory mechanisms of salinity and dietary lipid source on growth and hepatic lipid metabolism in spotted sea bass, integrating growth performance, liver morphology and transcriptomic analysis. A salinity level of 20‰ significantly enhanced growth performance but also increased hepatic lipid deposition. In contrast, the SO diets improved growth efficiency, although they were associated with greater lipid droplet accumulation. Transcriptomic analysis indicated that salinity primarily regulates steroid metabolism and fatty acid catabolism to support energy production and adapt to salinity changes. In contrast, dietary lipid source primarily affect lipid synthesis and catabolic metabolism. The interaction between salinity and lipid source regulated lipid metabolism primarily through the modulation of the Ppar pathway. These findings support precise nutritional strategies for spotted sea bass aquaculture across salinities: 1) Utilize SO as the primary dietary lipid source for fish cultured in both freshwater and seawater, adjusting dietary SO levels downward in freshwater to mitigate excessive lipid deposition; 2) Reduce reliance on FO by supplementing with functional additives (e.g., Ppar agonists) to counter the lipid-accumulating effects of SO.

## Data Availability

The data presented in the study are deposited in the NCBI repository, accession number PRJNA1304702.
