# Supplementary material for: Interactive effects of salinity and dietary lipid sources on growth, hepatic lipid metabolism, and transcriptomic profiles in spotted sea bass (Lateolabrax maculatus)
Source: Front Physiol. 2025 Aug 29;16:1655953. doi: 10.3389/fphys.2025.1655953 (PMC12426138; doi:10.3389/fphys.2025.1655953)
Supplement: Supplementary file 1 [file Supplementaryfile1.docx]

Table S1 Composition and nutrient levels of experimental diets (%)

| Items | Dietary Groups | |
| --- | --- | --- |
|  | Fish oil group | Soybean oil group |
| Fish meal | 25.00 | 25.00 |
| Chicken meal | 10.00 | 10.00 |
| Meat and bone meal | 8.00 | 8.00 |
| Corn gluten meal | 5.00 | 5.00 |
| Soybean meal | 18.00 | 18.00 |
| Soy protein concentrate | 8.50 | 8.50 |
| Fish oil | 12.00 | / |
| Soybean oil | / | 12.00 |
| Wheat four | 9.00 | 9.00 |
| Soybean phosphorus | 1.00 | 1.00 |
| Premix compound | 1.00 | 1.00 |
| Calcium hydrogen phosphate | 1.00 | 1.00 |
| Choline chloride | 0.50 | 0.50 |
| DL-methionine | 0.50 | 0.50 |
| L-lysine | 0.50 | 0.50 |
| Proximate composition (%) | | |
| Dry matter | 91.80 | 91.50 |
| Crude protein | 44.60 | 46.20 |
| Crude lipid | 15.10 | 15.30 |
| Crude ash | 10.00 | 10.50 |

Table S2 Primer sequences for qPCR

| Primers | Primer sequence (5' to 3') |
| --- | --- |
| *acc* | F: TCACAGACTACCGCTTCTTCATACG |
|  | R: TTACTGAACGCCACCTCCAACTC |
| *hmgcr* | F: CCTCCTCTATTCTTCGCCTCTTCTC |
|  | R: GCCACTCTGTCCCACTGTATCG |
| *hsd17b7* | F: CAGGCAGTTGCTCCATTT |
|  | R:GTCTCCAGCTCTGAGGTATT |
| *cyp24a1* | F: GTCTCCAGCTCTGAGGTATT |
|  | R:CTGTGCACAACTCCTCATAC |
| *cyp2r1* | F: CCAAAATGGGTGGGCTTCTC |
|  | R:TAGGCATCAACATAGTGGCGAG |
| *gpat3* | F: TACAAGAAAGGTCTGGAGAGCATCG |
|  | R:GGTTGGTTCTGGTGAGGAGGTTC |
| *dgat1* | F: CGAAGAGCGAGAAAAAGAAGC |
|  | R:TGACCACACACCAGTTGAGGA |
| *acsl4* | F: ATCGGCGAAGGTGTATATGTCTCC |
|  | R:CCACTCCTACTGCCTCCACAAC |
| *atgl* | F: CAGACAGGCAGGCAGACAGAC |
|  | R:CAAGACCACCTGAGACCGAGTTC |
| *acadl* | F: GGTTGACAGCGGCACTAAGG |
|  | R:AGCAGGCAAACGCACATCC |
| *hsl* | F: CCTGCTGGCTCCTAACAACCTG |
|  | R:TCCTCCACCACCGTCAGACTC |
| *acadsb* | F: atcacctgcttcatcgtggac |
|  | R:catacttgtacccatgaccga |
| *pltp* | F: CGCCAACTACAGAACCAACAGAAC |
|  | R:GAGCAGGCAGAAGAGACAGGAAG |
| *pparα* | F: AACCCGACTCTTATCCCTCC |
|  | R: CGTATCAACGCCACAGCAC |
| *18s rRNA* | F: GGGTCCGAAGCGTTTACT |
|  | R: TCACCTCTAGCGGCACAA |

*acadsb*: acyl-CoA dehydrogenase short/branched chain; *acc*: acetyl-coa carboxylase; *acsl4*: acyl-CoA synthetase long-chain 4; *atgl*: adipose triglyceride lipase; *cyp24a1:* 25-hydroxyvitamin D-24-hydroxylase; *cyp2r1,* vitamin D 25-hydroxylase; *dgat1*, diacylglycerol acyltransferase 1; *gpat3*: glycerol-3-phosphate acyltransferase 3; *hmgcr*: beta-hydroxy-beta-methylglutaryl Coaa reductase; *hsd17b7:* hydroxysteroid 17-beta dehydrogenase 7; *hsl*: hormone sensitive lipase; *pltp*: phospholipid transfer protein; *pparα*, peroxisome proliferator-activated receptor alpha.

Table S3 Pairs of representative genes related to lipid metabolism

| **Gene name** | **Log2FC** | **KEGG B class function annotation** | **Pathway** |
| --- | --- | --- | --- |
| **D1 vs D2** |  |  |  |
| *hsd17b7* | 1.55 | Lipid metabolism | ko00100//Steroid biosynthesis |
| *agpat9l* | 1.35 | Lipid metabolism | ko00561//Glycerolipid metabolism |
| *lss* | 1.34 | Lipid metabolism | ko00100//Steroid biosynthesis |
| *acadl* | -2.32 | Lipid metabolism | ko00071//Fatty acid degradation |
| *pdcl* | -1.54 | Lipid metabolism | ko00590//Arachidonic acid metabolism |
| *lpi1* | -1.38 | Lipid metabolism | ko00561//Glycerolipid metabolism |
| **D1 vs D3** |  |  |  |
| *hsd17b7* | 2.37 | Lipid metabolism | ko00100//Steroid biosynthesis |
| *cyp24a1* | 2.23 | Lipid metabolism | ko00100//Steroid biosynthesis |
| *lss* | 1.90 | Lipid metabolism | ko00100//Steroid biosynthesis |
| *ebp* | 1.78 | Lipid metabolism | ko00100//Steroid biosynthesis |
| *pltp* | 1.62 | Digestive system | ko03320//PPAR signaling pathway |
| *acsl4* | 1.54 | Lipid metabolism | ko00061//Fatty acid biosynthesis |
| *nsdhl* | 1.53 | Lipid metabolism | ko00100//Steroid biosynthesis |
| *sqle* | 1.48 | Lipid metabolism | ko00100//Steroid biosynthesis |
| *gcdh* | 1.02 | Lipid metabolism | ko00071//Fatty acid degradation |
| *cyp2r1* | -2.85 | Lipid metabolism | ko00100//Steroid biosynthesis |
| *acsl5* | -1.77 | Lipid metabolism | ko00061//Fatty acid biosynthesis |
| *acadl* | -1.66 | Lipid metabolism | ko00071//Fatty acid degradation |
| *pdcL* | -1.49 | Lipid metabolism | ko00590//Arachidonic acid metabolism |
| *acadsb* | -1.38 | Lipid metabolism | ko00071//Fatty acid degradation |
| *mgll* | -1.20 | Lipid metabolism | ko00561//Glycerolipid metabolism |
| *acot2* | -1.18 | Lipid metabolism | ko01040//Biosynthesis of unsaturated fatty acids |
| *acot1* | -1.10 | Lipid metabolism | ko01040//Biosynthesis of unsaturated fatty acids |
| *glpk2* | -1.02 | Lipid metabolism | ko00561//Glycerolipid metabolism |
| **D1 vs D4** |  |  |  |
| *pltp* | 1.46 | Endocrine system | ko03320//PPAR signaling pathway |
| *ebp* | 1.14 | Lipid metabolism | ko00100//Steroid biosynthesis |
| *acadl* | -1.90 | Endocrine system | ko03320//PPAR signaling pathway |
| *acsl5* | -1.72 | Endocrine system | ko03320//PPAR signaling pathway |
| *pdcL* | -1.47 | Lipid metabolism | ko00590//Arachidonic acid metabolism |
| *acadsb* | -1.47 | Lipid metabolism | ko00071//Fatty acid degradation |
| *mgll* | -1.38 | Lipid metabolism | ko00561//Glycerolipid metabolism |
| *acsl4* | 1.25 | Lipid metabolism | ko00061//Fatty acid biosynthesis |
| *acot1* | -1.24 | Lipid metabolism | ko01040//Biosynthesis of unsaturated fatty acids |
| *glpk2* | -1.10 | Endocrine system | ko03320//PPAR signaling pathway |
| *acox3* | -1.04 | Endocrine system | ko00071//Fatty acid degradation |
| **D2 vs D3** |  |  |  |
| *cyp24a1* | 2.19 | Lipid metabolism | ko00100//Steroid biosynthesis |
| *acsl4* | 1.20 | Lipid metabolism | ko00061//Fatty acid biosynthesis |
| *pltp* | 1.12 | Endocrine system | ko03320//PPAR signaling pathway |
| *dgat1* | 1.11 | Lipid metabolism | ko00561//Glycerolipid metabolism |
| *cyp2r1* | -2.69 | Lipid metabolism | ko00100//Steroid biosynthesis |
| *acadsb* | -1.22 | Lipid metabolism | ko00071//Fatty acid degradation |
| *acsl5* | -1.17 | Lipid metabolism | ko00061//Fatty acid biosynthesis |
| **D2 vs D4** |  |  |  |
| *cyp24a1* | 1.45 | Lipid metabolism | ko00100//Steroid biosynthesis |
| *dgat11* | 1.16 | Lipid metabolism | ko00561//Glycerolipid metabolism |
| *acadsb* | -1.28 | Lipid metabolism | ko00071//Fatty acid degradation |
| *ugt2a1* | -1.03 | Lipid metabolism | ko00140//Steroid hormone biosynthesis |
| **D3 vs D4** |  |  |  |
| *cyp2r1* | 2.20 | Lipid metabolism | ko00100//Steroid biosynthesis |
| *rarres3* | 1.25 | Lipid metabolism | ko00591//Linoleic acid metabolism |
| *hsd17b7* | -1.29 | Lipid metabolism | ko00100//Steroid biosynthesis |
| *lss* | -1.12 | Lipid metabolism | ko00100//Steroid biosynthesis |


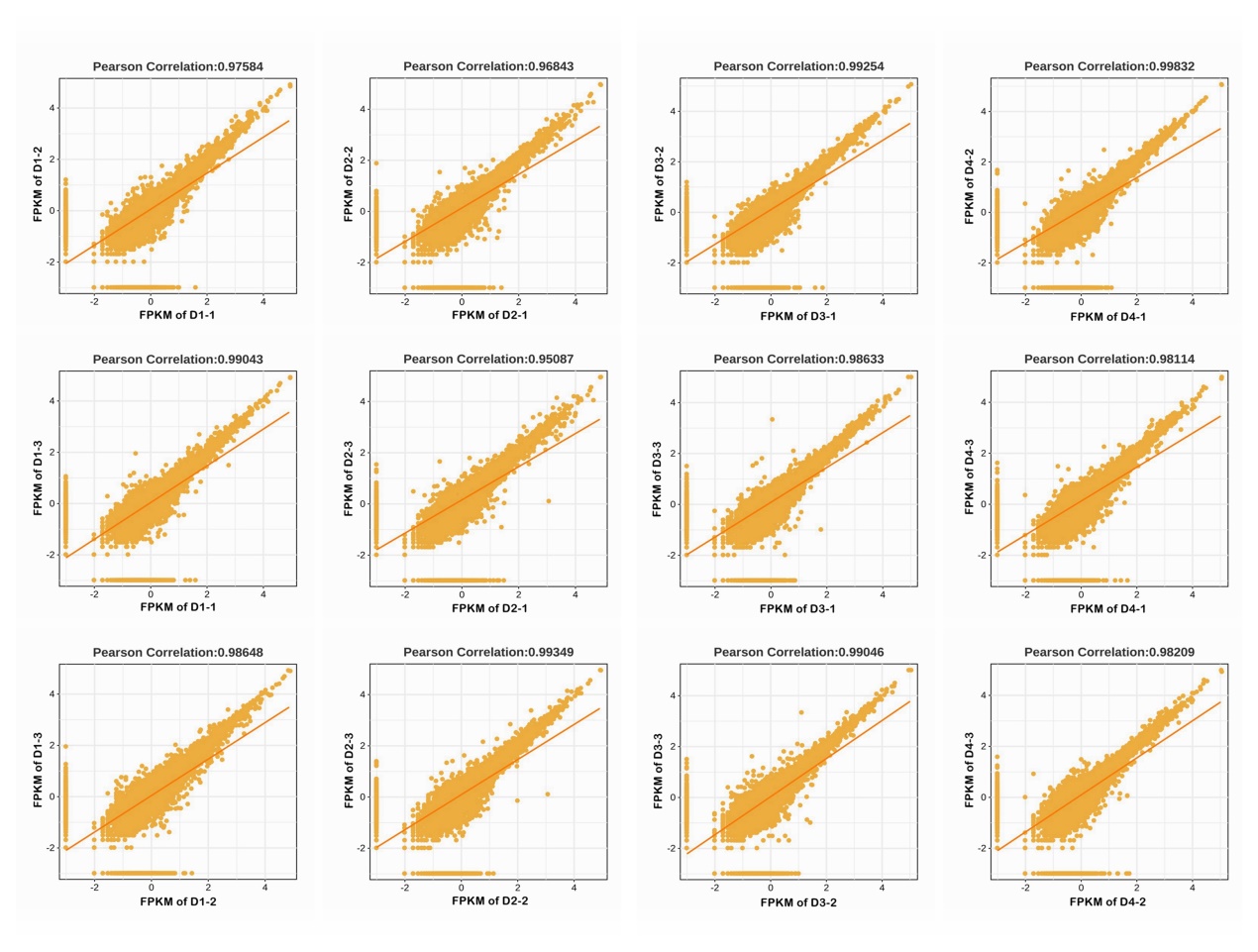


Figure S1 Pairwise sample relationship coefficient


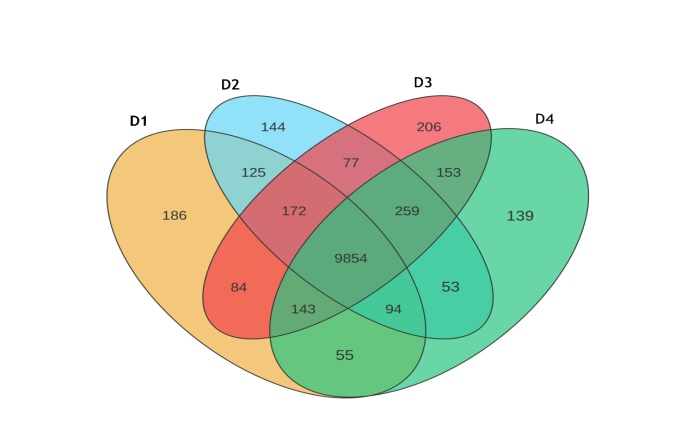

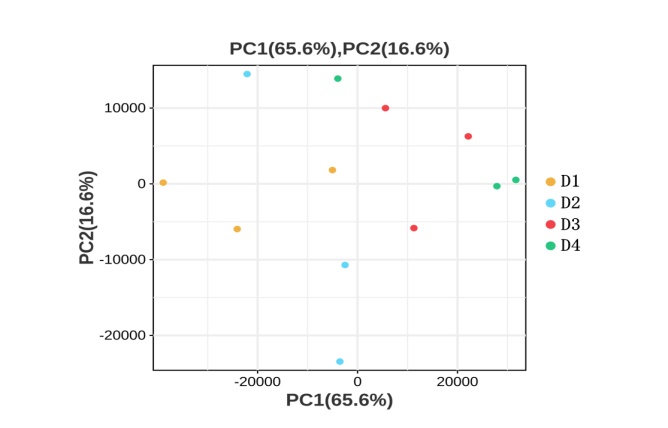


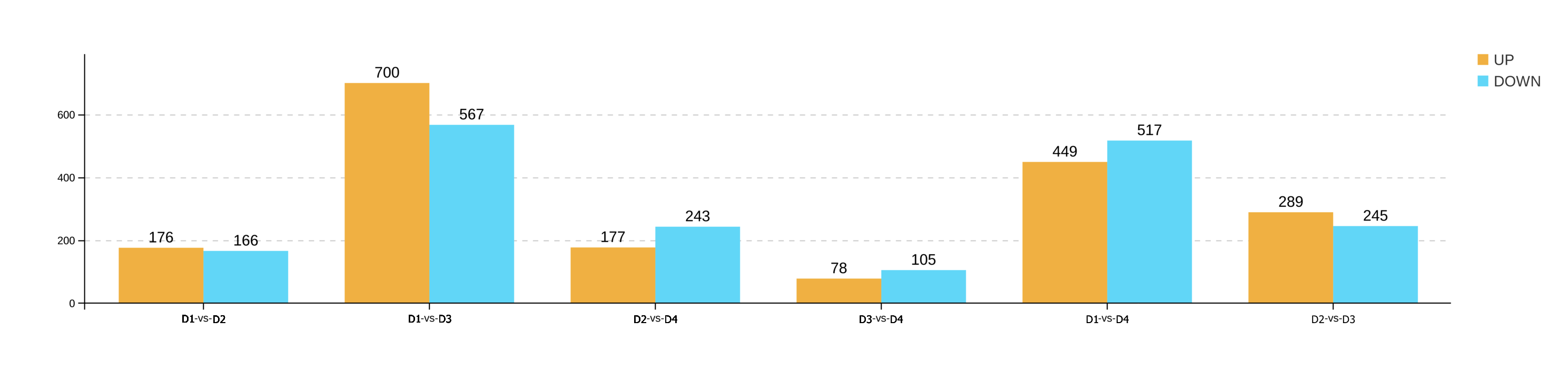


Figure S2 Principal component analysis of genes and DEGs comparison of Spotted sea bass among the different groups

Note: Groups D1 and D2 were fed FO- and SO-based diets, respectively, under freshwater conditions; groups D3 and D4 received the same diets under seawater conditions.
